# Imaging-based, non-radioactive measurements of natural killer cell activity provides comparable results as classic chromium release assays but with fewer cells and less labor

**DOI:** 10.1186/2051-1426-2-S3-P144

**Published:** 2014-11-06

**Authors:** Srividya Sundararaman, Kinga Karacsony, Diana Roen, Paul V Lehmann

**Affiliations:** 1 Cellular Technology Ltd., Shaker Hts., USA; 2 Pharmasan Labs Inc., USA

## Introduction

Natural Killer Cell activity has traditionally been assessed by detecting the lysis of tumor cells in The Chromium Release assay. This classic assay relies on radioactive labels, is laborious, and requires substantial quantities of patient blood. Moreover the Chromium Release assay provides low signal to noise ratios. We have developed an assay that can visualize individual target cells to detect cytolytic activity within a high signal to noise range, without involving radioactivity, via high-throughput imaging. We also developed a miniaturized version of this assay to measure NK activity with one tenth of the blood needed for a classic Chromium Release assay, and with less labor.

## Methods

The assay we developed is based on the imaging of individual fluorescence-labeled target cells. K562 tumor cells were used as targets, and peripheral blood mononuclear cells (PBMC) as effector cells. In addition to using inactivated PBMC, we also used IL-2 stimulated PBMC as effectors. When performing the assay in 96 well format, the PBMC were plated in serial dilution between 500,000 and 7,500 cells per well with 5,000 target cells per well. The Chromium Release assay was set up in parallel using identical conditions. Four hours later, the number of viable tumor cells was quantitated using a fluorescence capable ImmunoSpot^®^ Analyzer or the radioactivity released was measured. In a miniaturized version, one tenth of effector and target cells were plated in a Terasaki plate format.

## Results

The target cell visualization and Chromium Release assay in a 96-well format required the same number of cells and the results were comparable to each other. However, the signal to noise ratio was two orders of magnitudes higher for the target cell visualization assay, and required about a third of investigator's time invested. While, expectedly, percentage of killing for different donors was highly variable, the assay was highly reproducible for cryopreserved samples between multiple days. The assay results obtained in Terasaki plates, miniaturizing the assay tenfold, paralleled those of the 96-well format.

## Conclusion

We have demonstrated the feasibility of assessing NK function in a non-radioactive, high-throughput capable system which will benefit clinical immune monitoring. Signal to noise performance of the assay outperforms Chromium Release, as does the need for investigator effort. The miniaturized Terasaki format version of the assay should be of particular value when access to PBMC is limited, such as in pediatric, geriatric, and immune deficient populations.

